# Radiotherapy in oncological emergencies: fast-track treatment planning

**DOI:** 10.1186/s13014-020-01657-6

**Published:** 2020-09-10

**Authors:** Lukas Nierer, Franziska Walter, Maximilian Niyazi, Roel Shpani, Guillaume Landry, Sebastian Marschner, Rieke von Bestenbostel, Dominika Dinkel, Gabriela Essenbach, Michael Reiner, Claus Belka, Stefanie Corradini

**Affiliations:** Department of Radiation Oncology, University Hospital, LMU Munich, Marchioninistr. 15, 81377 Munich, Germany

**Keywords:** Emergency radiation treatment, Treatment planning on diagnostic CT, Fast treatment planning, Rapid planning, Emergency RT workflow

## Abstract

**Background and purpose:**

To report on our clinical experience with a newly implemented workflow for radiotherapy (RT) emergency treatments, which allows for a fast treatment application outside the regular working-hours, and its clinical applicability.

**Methods:**

Treatment planning of 18 emergency RT patients was carried out using diagnostic computed tomography (CT) without a dedicated RT simulation CT. The cone-beam CT (CBCT) deviations of the first RT treatment were analyzed regarding setup accuracy. Furthermore, feasibility of the “fast-track” workflow was evaluated with respect to dose deviations caused by different Hounsfield unit (HU) to relative electron density (rED) calibrations and RT treatment couch surface shapes via 3D gamma index analysis of exemplary treatment plans. The dosimetric uncertainty introduced by different CT calibrations was quantified.

**Results:**

Mean patient setup vs. CBCT isocenter deviations were (0.49 ± 0.44) cm (*x*), (2.68 ± 1.63) cm (*y*) and (1.80 ± 1.06) cm (*z*) for lateral, longitudinal and vertical directions, respectively. Three out of four dose comparisons between the emergency RT plan calculated on the diagnostic CT and the same plan calculated on the treatment planning CT showed clinically acceptable gamma passing rates, when correcting for surface artifacts. The maximum difference of rED was 0.054, while most parts of the CT calibration curves coincided well.

**Conclusion:**

In an emergency RT setting, the use of diagnostic CT data for treatment planning might be time-saving and was shown to be suitable for many cases, considering reproducibility of patient setup, accuracy of initial patient setup and accuracy of dose-calculation.

## Background and purpose

Ideally, oncological patients receive RT according to a fixed schedule, which allows for a clinical workflow of approximately 3–7 days from first patient contact to the beginning of treatment. This timeframe is necessary for a thorough assessment of the treatment indication, consideration of available diagnostic imaging data, coordination with other treating physicians, and the routine RT planning workflow. An RT workflow usually includes the acquisition of a planning CT in treatment setup patient positioning, target and organ at risk (OAR) delineation, dose prescription, treatment planning, and dose delivery. Approximately 3% of patients present with medical conditions requiring immediate RT [1]. These oncological emergencies are defined as “conditions arising from a reversible threat to an organ function, requiring radiation treatment within a few hours of diagnosis” [2]. In these cases, RT is indicated if no other measures are likely to have a similar rapid relief of symptoms [3].

As reported in a large pattern of care study of 3244 patients treated in Germany, Austria and Switzerland, the most common indications for emergency RT were acute spinal cord compression (42.3%), superior vena cava (SVC) syndrome (27.7%), bronchial obstruction (8.2%), tumor bleeding (8.5%), increased brain pressure (11.3%) or other not specified indications (2%) [1]. Regarding the efficacy of emergency RT, the same study reported response rates of 50% in patients treated for spinal cord compression, 70% in SVC syndrome, 70% in bronchial obstruction, 80% in tumor bleeding, 70% in brain pressure, and 80% in other indications [1, 4]. Most importantly patient outcome was significantly improved in patients with SVC syndrome if the time interval between referral and start of the emergency RT (hereinafter referred as preparation time) was less than 2 hours [1]. Therefore, immediate delivery of emergency RT is essential. This is usually guaranteed if emergency patients are referred to RT during regular working hours. However, emergencies frequently occur outside working hours and require immediate and adequate treatment with limited resources. To ensure access to emergency RT, we established a “fast-track” workflow that utilizes diagnostic CT images as planning CT and does not use immobilization devices or reference marks. We report on the feasibility of this “fast-track” treatment planning procedure. Furthermore, we investigated the feasibility of the new “fast-track” workflow in emergency RT with respect to the dosimetric impact of different tabletop geometries (curved vs. flat) and quantified dose calculation uncertainties induced by the uncertainty of HU to rED calibration curves of the diagnostic CT scanners.

## Materials and methods

We searched our database for RT treatments performed outside the working hours, between 01/16 and 08/19. Cases were included if no simulation CT was performed prior to the emergency RT.

### “Fast-track” workflow and setup deviations

**Fig. 1:**
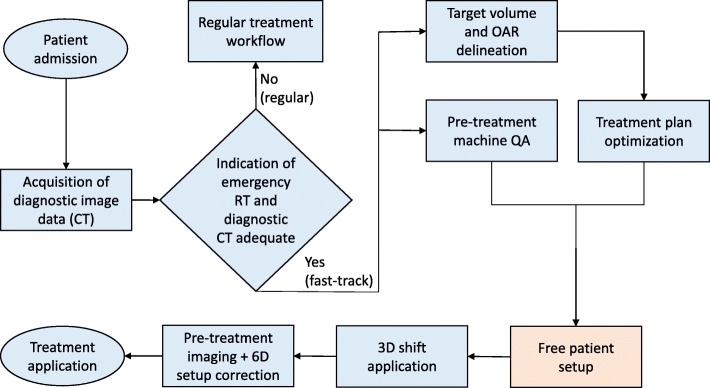
“Fast-track” emergency RT workflow

Patients presenting with oncological emergency indications are seen by an on-site radiation oncology resident. If the need for immediate treatment is verified by a certified radiation oncologist, the on-call radiotherapy technologists (RTT) and medical physicists are notified. According to our institutional standards, the on-call staff has to be on-site within 1 hour from notification. The on-site physician immediately starts the delineation of organs at risk and target volume using the available CT data. Ideally, the delineation process is completed by the time the on-call staff arrives. For dose calculation a default HU to rED conversion table was used for all diagnostic CT data. The treatment plan was optimized and approved. During treatment planning, the RTT prepared the linear accelerator (LINAC) in terms of machine warm-up, pre-treatment daily machine quality assurance and setup of immobilization devices. In order to perform pre-positioning of the patient prior to treatment delivery, palpable anatomic reference points (e.g. suprasternal notch) were used as virtual reference points to calculate relative vectors in the TPS to enable the calculation of couch shift values to the RT isocenter. In cases where no anatomic reference points were utilized, the physician performed a free pre-positioning of the RT isocenter. Patient positioning was corrected using CBCT. Finally, a robotic couch shift with 6 degrees of freedom (HexaPOD evo RT, Elekta AB, Stockholm, Sweden) was applied and treatment delivered. These translational couch correction values were recorded. Figure 1 shows a flowchart of the “fast-track” emergency RT workflow.

### Gamma index analysis and dose uncertainty

Exemplary patients were selected from the cohort to perform a dose comparison between the initial emergency RT plan on the diagnostic CT and the same treatment plan calculated on the dedicated planning CT. Patients were selected for this analysis, if the patient setup of the diagnostic CT was comparable to the patient setup of the planning CT. All cases with CTs at different breath-hold levels or anatomical changes (e.g. significant weight loss of patients) were excluded from this dose comparison.

The dose was calculated via Collapsed Cone algorithm, air cavities in the stomach, bowel or rectum were overridden with the rED of water, no beam attenuation through the couch tops was taken into account and one single default HU to rED calibration was used for all diagnostic and dedicated planning CTs. The isotropic dose grid voxels were 2.5 mm. The beam isocenter was placed according to an anatomic reference structure, dose distributions were registered rigidly via isocenter and a 3D γ-analysis was performed [5]. Both, whole dose distributions and cuboidal cut-out dose volumes inside the patient were compared to estimate the dose similarity without artifacts on the patient surface resulting from the rigid registration method. Moreover, a dose difference map was generated from the two dose volumes in order to visualize the dose comparison procedure.

Furthermore, HU to rED calibration curves of three different CT scanners of different vendors were measured at our institution and analyzed in order to estimate the dose uncertainty induced by the potential non availability of HU to rED calibration data for the diagnostic CTs. This is because uncertainties in rED estimation lead to dose calculation uncertainties. Measurements were performed with a tissue characterization phantom (Gammex Model 467, Melbourne, USA).

### Statistical analysis

Statistical analysis of mean values and variances of the setup correction values was performed via Welch-test, T-test, one-way analysis of variance (one-way ANOVA) and F-test. A significance level of α = 5% was used. Deviations in all translational directions were analyzed and total deviations were compared between patients who were positioned via anatomic reference point and free setup patients.

## Results

### Patients

Eighteen patients who were treated using the “fast-track” treatment planning procedure between 01/16 and 08/19 were included. Table 1 gives an overview of patient and treatment characteristics. The median age was 67 years (40–84 years), the most frequent indications were spinal cord compression or SVC syndrome. Emergency RT was applied in 5–12 fractions with single doses of 2 Gy – 4 Gy by the use of 3D conformal or intensity modulated radiotherapy (IMRT) treatment plans. The positioning of the patient before treatment delivery was performed using virtual reference points on palpable anatomic features in 8/18 cases. In 10/18 cases a free pre-positioning without any anatomical reference point was performed. In 12 out of 18 cases, the treatment plans were re-planned after proper CT simulation after 1–3 fractions during regular clinical routine. In cases where patient setup of the diagnostic CT was similar to patient setup on the treatment couch and dose plan quality could not be improved significantly (*n* = 6), the initial plan was continued throughout the treatment series.

**Table 1:** Patient and treatment characteristics

patient	age [yrs]	diagnosis	indication	reference point	fractionation	technique	re-planning [after fx-no.]
1	53	leukemia	leptomeningeal disease	na	5 × 4 Gy	3D	1
2	79	cervical cancer	bleeding	symphysis	10 × 3 Gy	IMRT	2
3	40	sarcoma	leptomeningeal disease	na	10 × 3 Gy	IMRT	2
4	67	bladder cancer	IVC syndrome	na	5 × 4 Gy	3D	na
5	60	leukemia	spinal cord compression	suprasternal notch	5 × 4 Gy	3D	1
6	40	sarcoma	spinal cord compression	suprasternal notch	5 × 4 Gy	3D	na
7	77	lymphoma	SVC syndrome	suprasternal notch	10 × 2 Gy	IMRT	3
8	83	breast cancer	SVC syndrome	suprasternal notch	9 × 3 Gy	3D	1
9	70	NSCLC	SVC syndrome	na	10 × 3 Gy	3D	na
10	66	NSCLC	SVC syndrome	suprasternal notch	10 × 3 Gy	3D	1
11	84	NSCLC	spinal cord compression	suprasternal notch	5 × 4 Gy	3D	na
12	59	multiple myeloma	nerval compression	na	10 × 3 Gy	3D	1
13	59	multiple myeloma	spinal cord compression	sternum	10 × 3 Gy	3D	2
14	60	multiple myeloma	leptomeningeal disease	na	7 × 4 Gy	3D	1
15	73	lymphoma	leptomeningeal disease	na	12 × 3 Gy	3D	1
16	79	lymphoma	spinal cord compression	na	5 × 4 Gy	3D	na
17	84	sarcoma	spinal cord compression	na	10 × 3 Gy	3D	1
18	58	prostate cancer	spinal cord compression	na	10 × 3 Gy	3D	na

*na* not applicable, *NSCLC* non-small-cell lung cancer, *IVC* inferior vena cava, *SVC* superior vena cava, *3D* 3D conformal, *IMRT* intensity modulated radiotherapy, *fx-no.* number of fractions

### Setup deviations

**Fig. 2:**
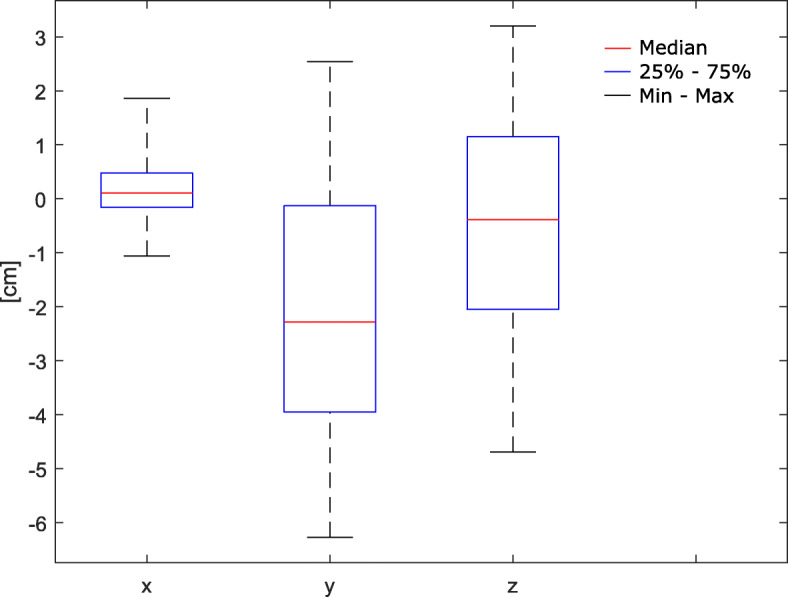
Boxplots of initial patient setup versus CBCT isocenter correction values in lateral (x), longitudinal (y) and vertical (z) direction of all patients (*n* = 18)

All CBCT total deviations (distance between RT isocenter and planned isocenter after initial patient setup) were smaller than 6.5 cm. Figure 2 shows boxplots of the CBCT correction values of the first treatment fraction of all patients (Table 2). The mean of the absolute correction values and their corresponding standard deviation were (0.49 ± 0.44) cm (*x*), (2.68 ± 1.63) cm (*y*) and (1.80 ± 1.06) cm (*z*) for lateral, longitudinal and vertical directions, respectively. Mean values of the absolute setup deviations in x-, y- and z-direction were significantly different (*p* < .001). The absolute setup deviations along the vertical axis (*p* < .001) and the longitudinal axis (*p* < .001) differed significantly from the corresponding positional errors along the lateral axis, while the deviations along the vertical and longitudinal did not differ significantly (*p* = .072). In contrast to the x-deviations (*p* = .354) and z-deviations (*p* = .368), the setup deviations in y-direction differed significantly from zero (*p* = .003). The mean vector deviation between setup (pre-imaging) and treatment isocenter (CBCT isocenter) was (3.55 ± 1.41) cm. Minimum and maximum total deviations were 0.94 cm and 6.41 cm, respectively. There was no significant difference between mean absolute correction values of patients with (x = (0.65 ± 0.54) cm, y = (2.23 ± 1.42) cm, z = (2.30 ± 1.11) cm) and without (x = (0.36 ± 0.28) cm, y = (3.03 ± 1.69) cm, z = (1.40 ± 0.83) cm) the use of a virtual reference point: x (*p* = .228), y (*p* = .325), z (*p* = .082).

**Table 2:** Setup correction values and the resulting vector (total) deviation: Initial patient setup vs. CBCT isocenter at first emergency RT fraction

patient	x (lateral) [cm]	y (longitudinal) [cm]	z (vertical) [cm]	total deviation [cm]
1	−0.16	− 2.82	−2.05	3.49
2	0.45	−1.44	−2.59	3.00
3	−0.04	−4.83	−2.50	5.44
4	0.97	2.54	−1.02	2.90
5	−0.56	−4.66	1.08	4.82
6	−0.06	−1.74	1.88	2.56
7	0.48	−1.39	−4.69	4.92
8	0.10	0.58	−1.89	1.98
9	0.35	−1.20	1.15	1.70
10	1.86	−3.22	3.20	4.91
11	0.60	0.84	1.77	2.05
12	−0.07	−0.13	0.93	0.94
13	−1.06	−3.95	−1.26	4.28
14	0.29	1.98	−3.07	3.66
15	0.11	−3.17	0.25	3.18
16	0.49	−4.36	−1.20	4.55
17	−0.43	−3.03	0.62	3.12
18	−0.66	−6.27	1.18	6.41
Mean	0.15	−2.02	−0.46	3.55

### Dose comparison / gamma passing rate

Table 3 shows results of the gamma index analysis for treatment plans which were re-planned after the emergency RT and which were suitable for dose comparison (*n* = 4). All other patients were not included in this analysis, as they did not receive an additional planning CT for re-planning due to a high consistency of diagnostic CT and CBCT, or because the patient diameter changed significantly. Mean values of the gamma passing rates for dose comparison of the whole patient volume and for the central volume were 93.3% (3 mm, 3%) and 88.8% (2 mm, 2%), and 98.2% (3 mm, 3%) and 96.5% (2 mm, 2%), respectively.

**Table 3:** 3D Gamma pass rates of the emergency RT treatment plan calculated on the diagnostic CT and the same plan calculated on a dedicated planning CT

pt.	γ (3 mm, 3%) whole volume [%]	γ (2 mm, 2%) whole volume [%]	γ (3 mm, 3%) central volume [%]	γ (2 mm, 2%) central volume [%]
1	94.9	89.8	99.2	96.0
2	91.1	88.0	100.0	99.2
3	89.3	81.2	94.0	92.0
17	97.9	96.0	99.4	98.7
Mean	93.3	88.8	98.2	96.5

**Fig. 3:**
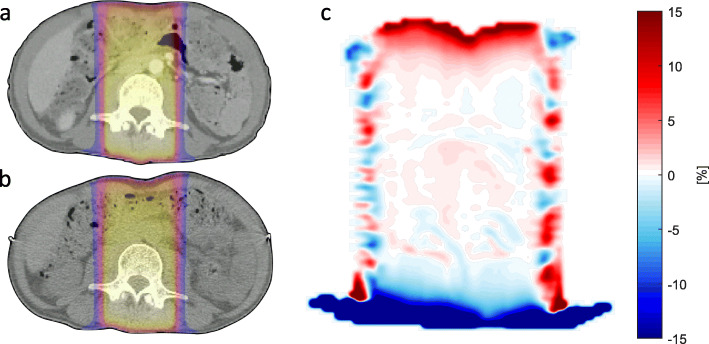
Exemplary 3D conformal emergency treatment plan dose distributions on axial slices of patient 1 (re-planned after 1st fraction) and the resulting dose difference map: **a** original plan calculated on diagnostic CT (no reference marks, curved CT couch surface, no immobilization devices), **b** same treatment plan calculated on the planning CT, which was acquired after the first fraction (rigid registration via isocenters; one vertebra was used as an anatomical reference structure for isocenter placement), **c** Dose difference map (cut off for doses < 10% of prescribed dose) with dose differences < 1% in the large central area

An example of the dose difference map is depicted in Fig. 3 and shows major deviations close to the patient surface in the beam directions, whilst in the central area most voxel doses differ less than 1%.

**Fig. 4:**
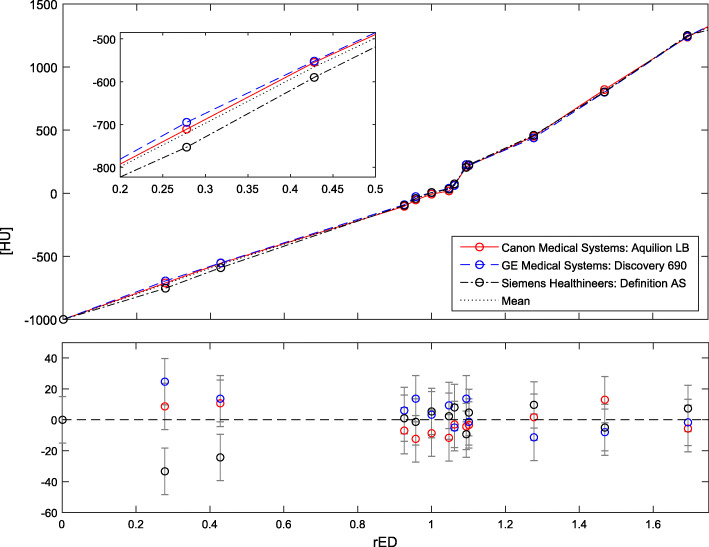
HU-rED calibration curves for different CT scanners from different vendors at our institution (120 kV abdominal protocol): *red:* Aquilion LB CT (Canon Medical Systems Corp., Otawara, JPN), *blue:* Discovery 690 PET-CT (GE Healthcare, Chalfont St Giles, GB), *black:* SOMATOM Definition AS (Siemens Healthineers, Erlangen, GER). Upper graph: HU-response curves and their mean, lower graph: deviations from the mean in absolute numbers

Figure 4 shows the measured HU-response curves and deviations from the mean value of CT scanners of different vendors at our institution. The maximum difference of rED between the three curves, and between the mean and the three curves was 0.054 and 0.033, respectively (linear interpolation between data points). Most parts of the response curves coincide well.

## Discussion

The main focus of this “fast-track” planning procedure was to improve practicability and preparation time of emergency RT outside regular working hours. The use of diagnostic CTs for treatment planning was time-efficient and overcame the need of the acquisition of an additional planning CT. After implementation of this new approach, the method has been successfully used in clinical practice and helped to keep the preparation time short. Overall, we found acceptable reproducibility and positional errors were within a manageable level.

Nevertheless, there remain substantial differences between a diagnostic and a RT planning CT: (1) the lack of reproducible patient positioning with immobilization devices, (2) missing reference marks, (3) the use of different tabletops, and (4) different HU calibration. Regarding patient positioning, no immobilization devices were used during diagnostic imaging. Nevertheless, patients were positioned using available immobilization devices during treatment delivery to mimic their position during the diagnostic CT. Due to the robotic couch it was possible to correct for any misalignments in six independent degrees of freedom. For cerebral RT, patients usually receive an individual thermoplastic mask for immobilization. Nevertheless, it is theoretically also possible to perform a whole-brain RT without the use of a thermoplastic mask with the assistance of a surface scanner [6–8]. A recent study proved the feasibility of this method in 30 patients and showed good clinical results with 93% of successful treatment delivery [9]. In the present study, no patients undergoing cerebral RT were included. Presumably, the total deviation of initial patient positioning strongly depends on the experience of the physician performing the initial patient setup. The setup deviations along the vertical axis and the longitudinal axis were significantly different from the corresponding positional errors along the lateral axis, where the mean absolute deviation from the CBCT isocenter was the smallest with only (0.49 ± 0.44) cm. This might be because the isocenter is frequently located in the midline of the patient and it is easier to find the correct lateral position of the isocenter as compared to the longitudinal and vertical axes. In contrast, finding the isocenter in longitudinal and vertical directions seems more challenging. There was no statistically significant difference between deviations of patients positioned with or without the use of virtual reference points. An evaluation of a larger cohort might be necessary to see if the free pre-positioning can be as accurate as the setup using anatomical reference points in the given setting. Although 78% of patients were pre-positioned too far in the cranial direction (valid for head first supine, HFS setup), experienced staff (physicans, RTTs) were able to position the patients of the present cohort within a reasonable range of correction values (compared to regular RT) of (3.55 ± 1.41) cm. However, no general rule can be defined for the threshold of acceptable total correction values in an emergency RT setting. In general, caution is advised with the handling of such comparably large setup correction values. However, results from a cohort of 1600 patients, of whom 190 received emergency RT, showed that near-miss incidents were not more frequent in emergency RT than regular RT [4]. On the other hand, the same study provided evidence that near-miss events that occur during emergency treatments on holidays or weekends tend to be of greater severity than those during the regular working week though. In the case of the new “fast track” approach, the involvement of physicians with limited clinical experience who have to deal with large correction values, could imply a higher risk for severe errors, for example due to a unrecognized geographical miss. Nevertheless, in the present study, no miss or near-miss incidents have been reported.

The attenuation properties of different tabletops can be easily taken into account via the treatment planning system (TPS). It is possible to either save the material composition and geometric properties of the RT treatment couch as a template, or a generic couch model can be used. The CT couch of the diagnostic CT can be easily removed in the TPS with regard to beam attenuation and dose calculation. Furthermore, different curvatures of the tabletop surface could potentially influence the dose distribution. However, this effect depends on the gantry angle and the monitor unit (MU) distribution of the intensity modulated radiation therapy (IMRT) segments or 3D conformal beams. Diagnostic CT tabletops are usually curved up on the side in contrast to the flat RT treatment tables and differences in the middle area of the table are smaller. Therefore, major dose deviations will occur at regions where diagnostic and treatment planning CT differ a lot due to different tabletop geometries. Nevertheless, in cases of spinal cord compression, SVC syndrome or bronchial obstruction (78.2% of emergency cases [1]), treatment plans usually have a high fluence in anterior-posterior or posterior-anterior (APPA) direction, which makes them robust in terms of different surface curvatures of the tabletop.

All emergency RT plans of the present gamma analyses had a high fluence in APPA direction. The differences in patient anatomy and registration uncertainty resulted in different build-up regions close to the skin. Obviously, these dose differences do not exist in reality, as they are artifacts of the rigid registration method (misalignment of patient surface). Assuming a gamma pass rate of 3 mm/ 3% > 95% and 2 mm/ 2% > 90%, only 1 out of 4 plan comparisons would have passed when considering the whole dose volume. However, when the different build-up regions were excluded from the gamma analysis, 3 out of 4 plans passed. This dose comparison shows that even if no CT scanner-specific HU-ED calibration data are available and the curvature of the treatment couch differs significantly, dose deviations can still remain within a clinically acceptable range. The present study provides evidence that the deviations in dose distribution may be considered clinically negligible in most emergency RT cases when treated with a 3D conformal plan with high fluence in APPA direction, as long as the patient anatomy does not change significantly between diagnostic CT acquisition and treatment application. In cases with major anatomical changes, it is likely that significant systematic dose deviations will occur. In the present study, no re-planning was performed in cases with a high anatomical similarity between diagnostic CT and setup CBCT, and therefore no planning CT was acquired which would allow a dose comparison. This means that the cases selected for dose comparison tend to provide a conservative estimation of dose deviation since only cases with low similarity were re-planned.

All TPS require rED information in terms of density correction (conventional dose calculation algorithms) or stopping power estimation (MC). An individual HU to ED calibration curve is acquired for each treatment planning CT scanner and saved in the TPS (or compared to the curve implemented in the TPS) [10–12]. When using diagnostic imaging, HU calibration data of the diagnostic CT scanners may not be available, which could result in dose calculation errors (larger variations in HU values result in a larger dosimetric error). This error increases with increasing tissue thickness and decreasing effective photon beam energy [10]. The HU values depend on the individual CT scanner, scanning protocol and tube voltage [13]. However, it has been shown that the tube voltage has no clinically relevant effect on the TPS dose calculation [13–15]. Tolerance levels of rED values can be defined with respect to a specific dosimetric effect in dose calculation that corresponds to a distinct tissue. Nakao et al. [14] showed that a tolerance level of 2% (local dose difference) corresponds to changes in rED of 0.044, 0.022 and 0.044 for lung, adipose / muscle and cartilage / spongy-bone tissue, respectively. In the present setting, the maximum difference of rED of the measured CT scanner response curves at our institution was 0.054, and the maximum difference from the mean was 0.033, which is within the range of the tolerance levels mentioned above. Additionally, the tolerance levels mentioned above are a worst-case scenario, since they are referred to 6 MV flattening filter free (FFF) photon beams. At our institution, the lowest clinically available photon energy is 6 MV with flattening filter (FF) which has a spectrum shifted to higher energies compared to 6 MV FFF due to beam hardening. Most parts of the calibration curves coincide far better than the tolerance level defined in the study by Nakao et al. [14]. Furthermore, the calibration curves of the different diagnostic in-house CT scanners can be integrated into to TPS and scanner-specific calibration curves used for treatment planning, if necessary. The information about the used CT scanner type can be retrieved from the Digital Imaging and Communications in Medicine (DICOM) metadata.

All uncertainties resulting from the use of diagnostic CT for treatment planning seem to be manageable in most cases of an emergency RT setting. Nevertheless, it has to be decided individually whether dose deviations are within an acceptable range. Special attention should be paid if major anatomical changes have occurred between the acquisition of the diagnostic CT and the RT treatment (e.g. weight loss might result in different beam attenuation and underestimation of dose in the TPS) or when more complex emergency treatment plans are necessary (higher degree of modulation, smaller field sizes or segments, or more evenly distributed beam angles in contrast to simple APPA 3D conformal treatment plans). Nevertheless, emergency patients are generally in poor general health condition and may not tolerate a prolonged treatment time on the RT treatment couch. Usually the focus is primarily on reduced beam-on time and plan robustness, rather than a highly sophisticated dose distribution. A limitation of the present study is that the validation of the reduction of preparation time is limited, since no time measurements were performed, which does not allow for a quantitative retrospective analysis of preparation time of the “fast-track” approach compared to the regular emergency RT workflow. Furthermore, this is a proof of concept study with limited statistical validity due to the small cohort size.

## Conclusion

A new workflow was implemented with the aim of shorter preparation time and a higher degree of flexibility in terms of RT emergency treatments. The first patients were successfully treated according to this “fast-track” approach and CBCT data of 18 patients were analyzed. In an emergency RT setting, the use of diagnostic CT data for treatment planning might be time-saving and was shown to be suitable for many cases, considering reproducibility of patient setup, accuracy of initial patient setup and accuracy of dose-calculation.

## Data Availability

Not applicable.
